# An Interventional Study on the Clinical Usefulness and Outcomes of Electroconvulsive Therapy in Medication-Resistant Mental Disorders

**DOI:** 10.7759/cureus.832

**Published:** 2016-10-17

**Authors:** Rameshwar S Manhas, Raheel Mushtaq, Shah Faisal Ahmad Tarfarosh, Sheikh Shoib, Mohammad Maqbool Dar, Arshad Hussain, Tabindah Shah, Sahil Shah, Mushbiq Manzoor

**Affiliations:** 1 Institute of Mental Health and Neurosciences, Postgraduate department of Psychiatry, Government Medical College, Srinagar, J & K, India; 2 Mood Disorder Clinic, Postgraduate Department of Psychiatry, Government Medical College, Srinagar, J & K, India; 3 Department of Neurology, Institute of Human Behaviour and Allied Sciences (IHBAS), Delhi, India; 4 Postgraduate Department of Psychiatry, Government Medical College, Srinagar, J & K, India; 5 Government Medical College, Srinagar, J & K, India; 6 Acharya Shri Chander College of Medical Sciences and Hospital, Sidhra, J & K, India; 7 Sher-i-Kashmir Institute of Medical Sciences Medical College, Srinagar, India

**Keywords:** electroconvulsive therapy, ect, interventional neurology, interventional psychiatry, healthcare technology, treatment resistance

## Abstract

**Background:**

Resistance to recommended medications has been an issue in dealing with a number of psychiatric ailments, and it is showing up as an ongoing challenge for contemporary mental health experts. Resistant psychiatric disorders not only increase the morbidity of patients suffering from such severe conditions but also intensify the problems of their caretakers. This has vigorously started to cause the costs to increase for healthcare services. Thanks to electroconvulsive therapy (ECT), we now have an effective method that is proving to be a fruitful final course of action in this micro-epidemic of resistant psychiatric diseases. However, the medical literature of case reports or studies in this niche is scarce. Also, no such comprehensive study has been carried out in the Southeast Asian region to date for the assessment of the effectiveness of electroconvulsive therapy in patients with medication-resistant psychiatric disorders.

**Aim:**

To assess the effectiveness of ECT in medication-resistant psychiatric patients at the post-ECT course, three-month follow-up, and six-month follow-up.

**Materials and methods:**

The study was a prospective and interventional study (without controls) conducted in the Institute of Mental Health and Neurosciences (IMHANS), Srinagar, India. Fifty-six patients with pharmacotherapy-resistant psychiatric disorders were included in the study. The patients were assessed at the end of the ECT course, at the three-month follow-up, and at the six-month follow-up by the Clinical Global Impression (CGI), Montgomery Asberg Depression Rating Scale (MADRS), Young Manic Rating Scale (YMRS) and the Yale-Brown Obsessive Compulsive Scale (YBOCS). Improvement was defined with the help of the CGI subscale by comparing the position of the patient at admission to the projected condition with ECT.

**Statistical analysis:**

Analysis of Variance (ANOVA) was used for analysis of the quantitative data. For the pair-wise comparison of the groups, the post hoc tests were used. Pearson’s chi-square test was used for analysis of qualitative data. A p-value of < 0.05 was considered to be statistically significant, and all the data analysis was done using SPSS Version 20.0.

**Results:**

The CGI scale revealed that statistically significant improvement occurred in patients at the end of ECT course, at the three-month follow-up as well as at the six-month follow-up.

**Conclusion:**

ECT should be used for the treatment of pharmacotherapy-resistant psychiatric patients and the benefits can be seen even six months after an ECT course completion. Further work in this field should focus on educating the general public about the usefulness of ECT in the treatment of resistant mental illnesses. The myths related to the so-called psychiatric assault from ECT should be removed.

## Introduction

The tale of mental anguish didn't always exist in the Kashmir valley in the present-day country of India. Violence, political changes, and numerous conflicts during the last 25 years in the Kashmir valley have led to a gradual rise in the prevalence of mental illnesses in the inhabitants of this valley [1-2]. Psychiatrists and psychologists of Kashmir have applied all recommended medical and psychotherapeutic approaches in the management of psychiatric disorders. However, it seems that just like the socio-political situation in the valley, which is resistant to any peaceful measure, more and more people are presenting psychiatric disorders resistant to currently approved medications – a phenomenon called treatment resistance [3].

While resistance to treatment badly influences the quality of life of patients, the electroconvulsive therapy (ECT) has the final solution. Not only is ECT becoming the psychiatrist’s magic wand in Western nations, it now forms an important part of treatment modalities used in Indian mental health institutions. Despite technological and economical advancement, India is still being referred to as a developing country, with ignorance and hesitation prevailing among the masses regarding the adoption of new and better forms of treatment. The people of India, in general, have surely not been able to completely appreciate the low side-effect profile and extremely high efficacy of ECT in treatment-resistant disorders; however, some indigenous mental health institutions are taking leaps forward in making full use of ECT by educating people about it [2-3]. Some institutions have gone even further to use ECT as a maintenance tool, with quite significant positive results on follow-up [4].

We are glad to be part of one of the pioneering institutions of India that use ECT, and we counsel people to take an active role in the decision-making process by offering this treatment methodology as an open and clearly defined treatment alternative (in some cases the only option of last resort, such as with treatment-resistant psychiatric disorders). To the best of our knowledge, there has been no comprehensive study done to date anywhere in the world that assesses the effectiveness of ECT in four groups of pharmacotherapy-resistant psychiatric patients, i.e., OCD, unipolar depression, bipolar affective disorder (BPAD) in depression, and BPAD in mania at a three-month and six-month post-ECT course.

This phenomenon of treatment resistance in psychiatry, analogous (but far more distinct in pathophysiology) to antibiotic resistance, has been described herein below for different subtypes of mental illnesses.

### Treatment resistance to depression

When at least two trials with antidepressants (adequate in terms of dosage, duration, and compliance) from different classes of drugs fail to produce a significant clinical improvement, the depression is labeled as treatment-resistant [5].

### Treatment resistance to mania

Patients with mania who fail to respond to a combination of two standard medications for a period of at least six weeks are labeled to be suffering from treatment-resistant mania [6].

### Treatment resistance to obsessive-compulsive disorder (OCD)

Patients of treatment-refractory obsessive-compulsive disorder (OCD) are those who have not responded to at least three therapeutic trials of selective serotonin reuptake inhibitors (SSRI) and selective norepinephrine reuptake inhibitors (SNRI), or with the usage of two or more atypical antipsychotics as treatment augmenting agents and behavioral therapy given while on the recommended dose of SSRI/SNRI [7].

## Materials and methods

### Setting

The study was conducted in the Institute of Mental Health and Neurosciences (IMHANS), Srinagar (India), which is an associated hospital of the Government Medical College (GMC), Srinagar, Kashmir (India). 

### Consent and approval

Ethical clearance for our study was obtained from the ethical committee of the GMC, Srinagar, and the informed consent was obtained from all the patients/their relatives (depending on the legal decision-making capacity).

### Study design

This study was a prospective interventional study (without a control group) conducted for a period of one year and eight months.

### Sample size

A total of 56 patients who suffered from psychiatric disorders resistant to pharmacotherapy were included in the study. After noting the sociodemographic information of each patient, they were assessed by the Clinical Global Impression (improvement subscale) one day after the last ECT, at the three-month follow-up, and at the six-month follow-up. Other scales used for assessment were the Montogomery Asberg Depression Rating Scale (MADRS), the Young Manic Rating Scale (YMRS), and the Yale-Brown Obsessive Compulsive Scale (YBOCS). These three scales were used one day before the first session of ECT, one day after the entire ECT course, and at the three- and six-month follow-up.

### Clinical Global Impression (CGI)

The Clinical Global Impression Scale consists of three different global measures:

1. The severity of the illness: assesses the current severity of the patient's symptoms (CGI-S). 2. Global improvement: compares the patient's baseline condition with his or her current state (CGI-I). 3. Efficacy index: compares the patient's baseline condition to a ratio of current therapeutic benefit and severity of side effects (CGI-E) [8].

### Montgomery-Asberg Depression Rating Scale (MADRS)

The Montgomery-Asberg Depression Rating Scale investigates the presence of affective, cognitive, behavioral, and somatic symptoms of depression. Ten symptoms are rated on a 0–6 scale, with possible scores of 0–60. The total score classifies patients according to levels of severity: normal or absent 0–6, mild 7–19, moderate 20–34, and severe 35–60 [9].

### Young Manic Rating Scale (YMRS)

The Young Manic Rating Scale is employed for the evaluation of mania symptoms. There are eleven items on the scale which are completed based on a patient's subjective report based on their clinical status from the previous 48 hours. These eleven items are increased mood, increased energy and locomotor activity, sexual desire status, sleeping, excitability, rate and amount of speech, language and thought disorder, thought content, destructive and aggressive behavior, appearance, and attitude. The maximum score for this test is 60 [10].

### Yale-Brown Obsessive Compulsive Scale (YBOCS)

The Yale-Brown Obsessive Compulsive Scale is a ten-item, clinician-rated instrument, each of which is scored from 0 to 4 to determine the severity of OCD and to monitor improvement during the treatment [11].

### Defining patient improvement

Improvement was defined from the CGI scale (improvement subscale) by comparing the position of the patient at admission to the projected condition with the therapy. If the patient attains a score of 1 or 2 on the CGI improvement (CGI-I) subscale, the patient is said to be improved.

### Socio-demographic and clinical data

A semi-structured case sheet was used for noting down the socio-demographic and clinical data of the patients.

### Inclusion criteria

Patients with pharmacotherapy-resistant psychiatric disorders (male and female).

### Exclusion criteria

1. Patients who did not give consent. 2. Patients who had never received a trial of pharmacotherapy. 3. Patients in whom general anesthesia was contraindicated. 4. Patients whose age was less than thirteen years.

### Process of ECT administration

A brief-pulse, bilateral, modified ECT was used on the patients. After receiving a signed informed consent from the patients/their relatives, the patients underwent physical examinations and necessary investigations along with pre-anesthetic check-ups by the consultants from the department of anesthesia. An effective ECT was considered to be a motoric seizure of not less than 15 seconds.

A total of 6-12 sessions of ECT were given to the patients. The sessions of ECT were continued until 1) the patient became fully asymptomatic and scored 1 or 2 on the CGI-I subscale, or 2) the patient showed no further improvement over two consecutive ECTs, or 3) consent for further continuation of ECT was not given, or 4) the patient completed a maximum of twelve sessions.

### Statistical analysis

Analysis of Variance (ANOVA) was used for analysis of the quantitative data. For the pair-wise comparison of groups, the post hoc tests were used. Pearson’s chi-square test was used for analysis of qualitative data. A p-value of < 0.05 was considered to be statistically significant, and all the data analysis was done using SPSS Version 20.0.

## Results

The mean age of all the studied patients was 39.6 (±11.76). Males constituted 51% of the group, and females were 48.2%. The major diagnoses were unipolar depression (53.6%) followed by BPAD in mania (19.7%). Table 1 shows age, sex, and clinical diagnosis of the studied group.

**Table 1:** Age, Sex, and Clinical Diagnosis of the Studied Group

Age (in years)	No. of Patients	Percentage
21-30	14	25%
31-40	15	26.8%
41-50	16	28.6%
51-60	10	17.8%
>60	1	1.8%
Mean = 39.6 (±11.76)		
Sex	No. of Patients	Percentage
Males	29	51.8%
Females	27	48.2%
Clinical Diagnosis	No. of Patients	Percentage
Unipolar Depression	30	53.6%
BPAD in Mania	11	19.7%
BPAD in Depression	10	17.8%
OCD	5	8.9%

Table 2 shows the number of ECTs administered to the patients. Thirty-four (68%) of the patients received 6-9 ECTs whereas 16 (32%) of patients received 10-12 ECTs. The mean number of ECTs received is 8.22 (±2.073).

**Table 2:** Number of ECTs Received by Patients

No. of ECTs	No. of Patients	Percentage
6-9	34	68%
10-12	16	32%
Total	50	100%
Mean=8.22 (±2.073)		

Table 3 shows the CGI-improvement of the patients overall at the end of the ECT course, at the three-month follow-up, and at the six-month follow-up. At the end of the ECT course, 39 (78%) patients were improved. At the three-month follow-up after the ECT course, 32 (64%) patients were improved. At the six-month follow-up after the ECT course, 29 (67.4%) patients were improved. The p-value is 0.218 which is insignificant.

**Table 3:** Global Improvement of Overall Studied Patients

	Total No. of Patients	Improved	Not Improved	Chi-Square	P-Value
End of ECT Course	50 (100%)	39 (78%)	11 (22%)	2.511	0.28
Three-Month Follow-up	50 (100%)	32 (64%)	18 (36%)		
Six-Month Follow-up	43 (100%)	29 (67.4%)	14 (32.6%)		

Table 4 shows the CGI-improvement of the patients with bipolar depression (BPAD) at the end of the ECT course, at the three-month follow-up, and at the six-month follow-up. At the end of the ECT course, 7 (77.8%) patients were improved. At the three-month follow-up after the ECT course, 6 (66.7%) patients were improved. At the six-month follow-up after the ECT course, 6 (75%) patients were improved. The p-value is 0.859 which is insignificant.

**Table 4:** Global Improvement in Patients with BPAD in Depression

	Total No. of Patients	Improved	Not Improved	Chi-Square	P-Value
End of ECT Course	27 (100%)	21 (77.8%)	6 (22.2%)	1.438	0.859
Three-Month Follow-up	27 (100%)	17 (66.7%)	10 (33.3%)		
Six-Month Follow-up	21 (100%)	6 (75%)	6 (25%)		

Table 5 shows the CGI-improvement of the patients in unipolar depression at the end of the ECT course, at the three-month follow-up, and at the six-month follow-up. At the end of the ECT course, 21 (77.8%) patients were improved. At the three-month follow-up after the ECT course, 17 (63%) patients were improved. At the six-month follow-up after the ECT course, 15 (71.4%) patients were improved. The p-value is 0.487 which is insignificant.

**Table 5:** Global Improvement in Patients with Unipolar Depression

	Total No. of Patients	Improved	Not Improved	Chi-Square	P-Value
End of ECT Course	9 (100%)	7 (77.8%)	2 (22.2%)	1.4	0.487
Three-Month Follow-up	9 (100%)	6 (63%)	3 (37%)		
Six-Month Follow-up	8 (100%)	6 (71.4%)	2 (28.6%)		

Table 6 shows the CGI-improvement of patients with BPAD in mania at the end of the ECT course, at the three-month follow-up, and at the six-month follow-up. At the end of ECT course, 8 (88.9%) patients were improved. At the three-month follow-up after the ECT course, 8 (88.9%) patients improved. At the six-month follow-up after the ECT course, 7 (77.8%) patients were improved. The p-value is 0.746 which is insignificant.

**Table 6:** Global Improvement in Patients with BPAD in Mania

	Total No. of Patients	Improved	Not Improved	Chi-Square	P-Value
End of ECT Course	9 (100%)	8 (88.9%)	1 (11.1%)	0.587	0.746
Three-Month Follow-up	9 (100%)	8 (88.9%)	1 (11.1%)		
Six-Month Follow-up	9 (100%)	7 (77.8%)	2 (22.2%)		

Table 7 shows the CGI-improvement of the patients with OCD at the end of the ECT course, at the three-month follow-up, and at the six-month follow-up. At the end of the ECT course, 3 (60%) patients were improved. At the three-month follow-up after the ECT course, only 1 (20%) patient was improved. At the six-month follow-up after the ECT course, 1 (20%) patient was improved. The p-value is 0.301 which is insignificant.

**Table 7:** Global Improvement in Patients with OCD

	Total No. of Patients	Improved	Not Improved	Chi-Square	P-Value
End of ECT Course	5 (100%)	3 (60%)	2 (40%)	2.4	0.301
Three-Month Follow-up	5 (100%)	1 (20%)	4 (80%)		
Six-Month Follow-up	5 (100%)	1 (20%)	4 (80%)		

Table 8 shows the mean MADRS scores in unipolar and bipolar depression, the mean YMRS scores in BPAD in mania, and the mean (SD) YBOCS scores in OCD patients (at pre-ECT, end of ECT, three-month follow-up, and six-month follow-up).

**Table 8:** Mean MADRS scores in Unipolar and Bipolar Depression, Mean YMRS Scores in BPAD in Mania, and Mean (SD) YBOCS Scores in OCD Patients Shown at pre ECT (M1), end of ECT course (M2), at the three-month follow-up (M3), and at the six-month follow-up (M4).

	Mean MADRS Score in Unipolar Depression	Mean MADRS Score in Bipolar Depression	Mean YMRS Score in BPAD in Mania	Mean YBOCS Score in OCD
Pre-ECT (M1)	41.60+4.88 (n=30)	41.50+1.07 (n=10)	50.09+3.936 (n=11)	28.60+3.71 (n=5)
End of ECT Course (M2)	11.41+8.13 (n=27)	9.11+7.11 (n=9)	12.33+8.10 (n=9)	16.10+8.87 (n=5)
Three-Month Follow-up (M3)	15.59+12.38 (n=27)	13.11+13.53 (n=9)	12.44+10.643 (n=9)	24.60+10.66 (n=5)
Six-Month Follow-up (M4)	12.62+9.93 (n=21)	17.00+14.07 (n=8)	14.22+9.39 (n=9)	24.40+11.08 (n=5)

Table 9 shows the comparison of mean MADRS scores in patients with unipolar depression and bipolar depression at pre-ECT (M1) with the end of the ECT course (M2), with the three-month follow-up (M3), and with the six-month follow-up (M4). The difference of the mean MADRS in unipolar and bipolar depression and the mean YMRS scores in mania patients between pre-ECT (M1) and at the end of the ECT course (M2), between pre-ECT and the three-month follow-up (M3), and between pre-ECT and the six-month follow-up (M4) is statistically significant (p-value  ≤ 0.0001). The comparison of the mean YBOCS scores of patients with OCD at pre-ECT with the end of the ECT course, with the three-month follow-up, and with the six-month follow-up is shown. The difference of the mean YBOCS scores between pre-ECT and at the end of the ECT course is the only significance (p-value  ≤ 0.0001).

**Table 9:** Comparison of Mean MADRS Scores in Unipolar and Bipolar Depression, YBOCS Score in OCD, and YMRS Score in Mania Patients Shown at pre-ECT (M1) with: end of ECT course (M2), three-month follow-up (M3), and six-month follow-up (M4).

	Unipolar Depression		Bipolar Depression		OCD		BPAD in Mania	
Comparison	Mean Difference	P-Value	Mean Difference	P-Value	Mean Difference	P-Value	Mean Difference	P-Value
MI V M2	30.193	≤0.0001	32.389	≤0.0001	12.50	0.044	37.758	≤0.0001
M1 V M3	26.007	≤0.0001	28.389	≤0.0001	4.00	0.496	37.646	≤0.0001
M1 V M4	28.981	≤0.0001	24.500	≤0.0001	4.20	0.475	35.869	≤0.0001

## Discussion

**Figure 1:**
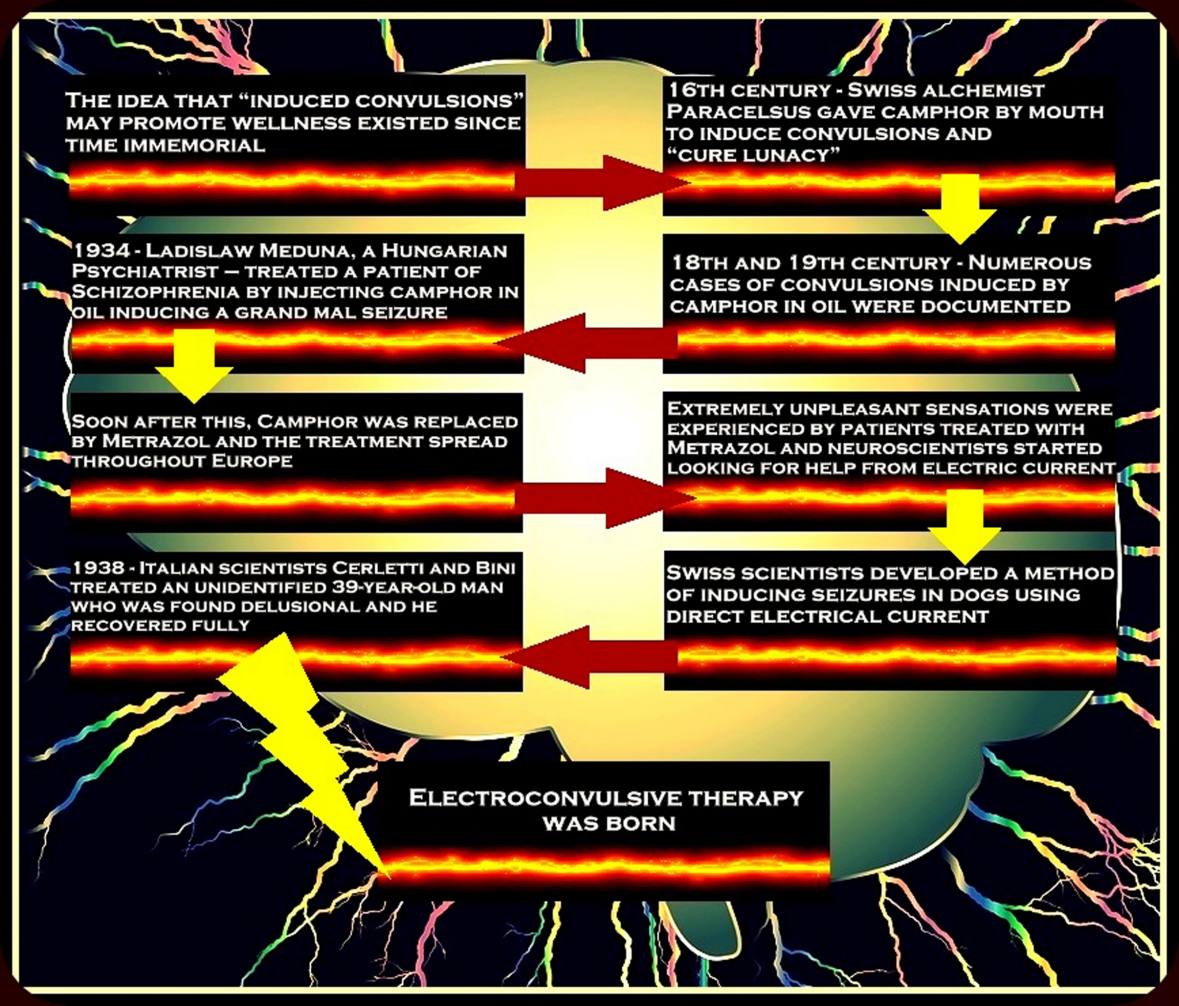
The Birth of Electroconvulsive Therapy A summary of historical events leading to the actual usage of electric current over the human scalp in clinical practice [12].

Despite the concept behind "inducing convulsions" in treating mental disorders being very old, its active usage in clinical practice started relatively very late. Figure 1 summarizes the historical timeline of the birth of ECT [12].

ECT is considered a first-line treatment when:

1. Psychiatric or medical factors require a quick and robust clinical response, 2. ECT poses less risk to a patient than pharmacotherapy (e.g., in elderly patients or during pregnancy), 3. There is a clear history of pharmacotherapy resistance or a history of favorable response to ECT, 4. The patient prefers being treated with ECT rather than medication [12].

In our study, we have made a successful attempt to bring the above-mentioned third indication of ECT, i.e., medication resistance, into the limelight. The mean age of our study population was 39.6 (± 11.76) years, approximately ten years younger than people receiving ECTs in western nations [13]. The other important findings of the study can be described under the following subheadings.

### Clinical Global Impression improvement (CGI-I) at the end of an ECT course

In our study, 50 patients out of the total of 56 patients (89.28%) completed the course of ECT. It is worth mentioning that 78% of those 50 study participants were reported as improved (according to the CGI-I scale). This finding of our study is in accordance with the study conducted by Moksnes et al., (2010) who found clinical improvement at the end of an ECT course in 85.1% of the patients receiving electroconvulsive therapy [13]. In our patients of BPAD in mania, we saw improvement in 88.9% at the end of the ECT course. The potent, anticonvulsant characteristics of ECT do explain this high effectiveness of ECT in our mania patients. Besides the mood stabilizers like valproate and carbamazepine, that are used to treat mania and have proven anticonvulsant properties, they are an effective management modality in medication-responsive patients with mania [12].

The patients from the unipolar depression group who were improved at the end of the ECT course were 77.8%. Although our study was without control groups, there are some controlled studies in the medical literature that suggest that about 70% of patients who fail to respond to antidepressant medications may respond positively to ECT [14]. Nevertheless, this finding is in contrast to a study conducted by Meddaa et al., (2009) that showed much more improvement in patients with unipolar depression compared to the findings of our study [15]. The fact that in our study we included patients with only pharmacotherapy-resistant unipolar depression might explain this difference in response as the response rate of medication-resistant patients to ECT might be lower as compared to medication-responsive patients [16].

In the BPAD in depression group, 77.8% of the patients were improved at the end of ECT course. This finding of our study is supported by the study conducted by Dabrowski et al., (2012). This study reveals that about 73% of the depressive patients with BPAD in depression were fully improved following the course of ECT [17].

All around the globe, OCD is quite difficult to treat. It was a pleasant surprise for us to find that 60% of our OCD patients showed improvement at the end of the ECT course. A thorough review of the literature regarding the management of OCD shows that the use of ECT in medication resistant OCD is quite sparse. Only few case reports showing the efficacy of ECT in OCD have been reported in the medical literature (to the best of the knowledge of all authors). However, the primary indications in all those case reports for the use of ECT would be OCD with severe depression [7]. The results of our study suggest that ECT is effective in controlling the obsessive and compulsive symptoms of medication-resistant OCD. We believe that the mechanism for this finding might be that ECT causes an increase in the serotonergic activity of the brain [18-19]. Dell'Osso et al., (2005) stated that this treatment modality has a good anti-obsessional effect which might explain the improvement of symptoms in OCD in our study population [20].

It was also observed that at the end of the ECT course, a significant decrease occurred in the mean YBOCS scores of OCD patients, mean MADRS scores in depressive disorders and BPAD with depression, and YMRS scores in mania patients (p-value is ≤ 0.0001). This finding is supported by findings of a handful of studies in which a significant decrease of mental symptoms occur in different psychiatric disorders at the completion of an ECT course [20-21].

### CGI-I at the three-month follow-up

Out of the 50 patients completing the course of ECT, 64% of patients were improved at three months ECT follow-up. To the best of the knowledge of the authors, no study has been done that assessed improvement in all psychiatric disorders at three months ECT follow-up. The findings of our study revealed that 88.9% of mania patients continued to remain improved at three months ECT follow-up. The finding of our study suggests that the efficacy of ECT in manic patients was sustained up to three months after the end of the ECT course. In unipolar depression patients, 63% remained improved at the three-month follow-up ECT. This study also shows a decrease of the mean MADRS score at three months (p-value ≤0.0001). This finding is supported by McCall et al., (2001), who claimed improvement in symptoms of depression at the first-month and additional improvement at the third-month post-ECT [22]. However, the mean difference in the MADRS score between the end of the ECT course and at the three-month follow-up is insignificant, which is in accordance with Huuhka et al. (2004) [23]. The patients with BPAD in depression who remained improved at three months after ECT were 66.7%. It was interesting to note that the improvement in bipolar depression patients at the three-month follow-up ECT (i.e. 66.7%) is nearly same as in unipolar depression (i.e., 63%). Dierckx et al., (2012) and Daly et al., (2001) also found that patients with bipolar and unipolar depression did not differ in rates of response to ECT [24-25]. Further, there were only 20% of OCD patients who improved at the three-month ECT follow-up. There was very little improvement in OC symptoms at pre-ECT levels and at the three months after ECT (as there was no subsequent change in YBOCS scores during this interval).

### CGI-I at the six-month follow-up

Out of 56 patients at the start of the study, 43 patients were followed up to six months after ECT. Patients who remained improved were 67.4%. This finding of our study is in contrast to Rey et al., (1997), who noted that 53% of their patients improved at the six-month ECT follow-up [26]. The difference in the results might be due to the fact that in our study, the maximum number of patients (88.4%) who followed to the six-month follow-up ECT were of mood disorder (47.5%). Mood disorders are known to have a high rate of response to ECT when compared to other psychiatric disorders [27-28]. There was also subsequent improvement at the end of the six-month ECT follow-up in each group of patients, i.e., in mania patients (77.8%), in unipolar depressive disorders (77.8%), in BPAD in depression (75%), and in OCD patients (20%). There was also improvement in the mean MADRS in both unipolar and bipolar depression, and the YMRS in mania patients. However, improvement in the YBOCS score was modest. Similar findings have also been reported earlier [27–29]. The comparison of improvement between the end of an ECT course, at the three-, and at the six-month follow-up ECT of the whole group and individual psychiatric disorders is insignificant, which suggests that change in the level of improvement up to six months is insignificant, and improvement with ECT is maintained up to six months after an ECT course. Maintenance of such high rates of improvement at three months and six months after an ECT course might be due to the fact that we were using psychotropics during and after the course of ECT, which might have augmenting effects and help in preventing relapses in patients treated with ECT. It is well-known that psychotropics are safer in combination with ECT over short- or long-term management of psychiatric disorders [27-28]. However, in OCD patients, improvement was only in 20% of the patients at the three- and six-month follow-up ECT, suggesting that ECT might not be an effective treatment for OCD. YBOCS scores at the pre-ECT level and at six months after ECT were almost the same, and ECT is not an effective long-term means of management of OCD (p=0.475). This finding of our study is supported by Khanna et al., (1988) who found that at six months after the initial treatment with ECT, the OC symptoms returned to the pre-ECT level [29].

### Limitations

1. The size of the study group was small. 2. The study was conducted in one hospital only.

## Conclusions

ECT is an effective treatment for medication-resistant mental disorders, with effectiveness lasting up to six months in the post-ECT follow-ups in the groups except in the OCD group. Future clinical research work should lay emphasis on the formulation of standardized guidelines regarding the course and duration of ECT sessions in different mental illnesses. It should also focus on the enhancement of other device-based therapies similar to (but more efficacious than) the current system of ECT delivery for the management of treatment-resistant mental disorders. Creating awareness for the general public about the usefulness of ECT and motivating healthcare researchers to work in this exciting field could very well prove fruitful in the management of difficult-to-treat neuropsychiatric illnesses.
